# Neuro-Nutritional Approach to Neuropathic Pain Management: A Critical Review

**DOI:** 10.3390/nu17091502

**Published:** 2025-04-29

**Authors:** Giorgia Cominelli, Francesca Sulas, Daniela Pinto, Fabio Rinaldi, Gaia Favero, Rita Rezzani

**Affiliations:** 1 Anatomy and Physiopathology Division, Department of Clinical and Experimental Sciences, University of Brescia, 25123 Brescia, Italy; giorgia.cominelli@unibs.it (G.C.); francesca.sulas@unibs.it (F.S.); gaia.favero@unibs.it (G.F.); 2 Human Microbiome Advanced Project Institute, 20129 Milan, Italy; dpinto@giulianipharma.com (D.P.); fabio.rinaldi@studiorinaldi.com (F.R.); 3 Interdepartmental University Center of Research “Adaption and Regeneration of Tissues and Organs-(ARTO)”, University of Brescia, 25123 Brescia, Italy; 4 Italian Society for the Study of Orofacial Pain (Società Italiana Studio Dolore Orofacciale–SISDO), 25123 Brescia, Italy

**Keywords:** pain/neuropathic pain, inflammation, oxidative stress, diet, melatonin

## Abstract

Pain is a significant global public health issue that can interfere with daily activities, sleep, and interpersonal relationships when it becomes chronic or worsens, ultimately impairing quality of life. Despite ongoing efforts, the efficacy of pain treatments in improving outcomes for patients remains limited. At present, the challenge lies in developing a personalized care and management plan that helps to maintain patient activity levels and effectively manages pain. Neuropathic pain is a chronic condition resulting from damage to the somatosensory nervous system, significantly impacting quality of life. It is partly thought to be caused by inflammation and oxidative stress, and clinical research has suggested a link between this condition and diet. However, these links are not yet well understood and require further investigation to evaluate the pathways involved in neuropathic pain. Specifically, the question remains whether supplementation with dietary antioxidants, such as melatonin, could serve as a potential adjunctive treatment for neuropathic pain modulation. Melatonin, primarily secreted by the pineal gland but also produced by other systems such as the digestive system, is known for its anti-inflammatory, antioxidant, and anti-aging properties. It is found in various fruits and vegetables, and its presence alongside other polyphenols in these foods may enhance melatonin intake and contribute to improved health. The aim of this review is to provide an overview of neuropathic pain and examine the potential role of melatonin as an adjunctive treatment in a neuro-nutritional approach to pain management.

## 1. Introduction

Pain is a global health priority, as it is known that one in five adults suffers from pain [1]. The prevalence of chronic pain varies between 11% and 40%, with a study conducted by the US Centers for Disease Control and Prevention (CDC) estimating the point prevalence at 20.4% [2]. Chronic pain is defined as pain lasting for more than three months [3,4] and is classified as primary, occurring in the absence of identifiable underlying diseases, and secondary, arising from other conditions such as tumors, inflammatory disorders, or autoimmune diseases [5,6]. This new classification has opened up a useful worldwide debate [7]. It has been demonstrated that multiple biological and psychosocial variables contribute to differences in pain, including gender, age, genetic susceptibility, and neurological correlates [8,9].

Various underlying causes, symptoms, and neurobiological mechanisms differentiate nociceptive, inflammatory, and neuropathic pain [10,11]. Neuropathic pain is a persistent or intermittent spontaneous pain characterized by an increased response to mechanical and thermal stimuli, whether innocuous or noxious, that arises from lesions to the somatosensory nervous system of peripheral or central origin [12,13]. It involves ectopic activity in sensorial fibers and an imbalance in the relationship between inhibitory and excitatory neurotransmitters [14,15]. It is also associated with neuroinflammation, glial cell activation, and nitrooxidative stress, which play a pivotal role in its instauration and maintenance [13,14]. Neuroinflammation and oxidative stress influence each other in a vicious cycle [15,16].

Epidemiological studies have suggested that neuropathic pain affects approximately 7–10% of the general population [14]. Despite the availability of pharmacological treatments, only partial pain relief can be achieved, and the traditional medications are associated with various adverse effects, which affect patients’ quality of life [15,16].

The International Association for the Study of Pain (IASP) defined nociceptive pain as “*pain that arises from an actual or threatened damage to non-neural tissue and is due to the activation of nociceptors*” [17,18,19]. Nociceptive pain is the most common type of acute pain; however, unfortunately, it may become chronic [20,21]. Nociceptive pain arises from actual or threatened damage to non-neural tissue due to the activation of the nociceptors [22], which are specialized peripheral nervous system (PNS) receptors that respond to one or more types of noxious stimuli of mechanical, thermal, or chemical origin [20].

It is important to distinguish between “pain” and “nociception”: pain encompasses a subjective and conscious perception, whereas nociception refers to the neuronal mechanisms that encode noxious stimuli [20,23]. Pain perception and the nociceptive system are integrated parts of the nervous system that allow an individual to interact with the environment and react accordingly to the received stimuli. In most cases, the purpose of feeling pain is to protect a subject from further harm and facilitate healing [20,21]. However, when pain becomes a pathological condition, it severely affects the quality of life. The protective nature of pain disappears, and it induces painful sensations [24].

Neuropathic pain is not caused by the activation of nociceptors but is due to damage to or disease of the somatosensory nervous system [20,25]. The complexity and evolving nature of neuropathic pain, characterized by diverse underlying mechanisms that change over time, have posed more challenges in its treatment [26]. Considering these challenges, non-pharmacological therapies may serve as valuable adjuncts in pain management and could enhance the effectiveness of pharmacological strategies [27,28,29,30,31,32].

In the field of pain management, the concept of pain catastrophizing is a central focus of study [33]. This construct was originally introduced by Ellis (1962) [34] and is related to a set of exaggerated and negative cognitions and emotions during actual or perceived painful stimulation [9]. Pain catastrophizing refers to a dysfunctional and negative mentality when coping with actual or anticipated pain. It comprises three distinct but related subscales: rumination (“I always worry about whether it will end”), magnification (“It is awful, and I fear that it will never get better”), and helplessness (“I feel like I can’t go on”) [35,36,37]. The relative contributions of these three separate subscales to the pain outcomes associated with painful post-traumatic trigeminal neuropathy are limited; thus, focusing on pain catastrophizing in managing painful neuropathy should be considered. To date, unfortunately, there are limited clinical trial data in the field of neuropathic pain focusing on psychological interventions targeting pain catastrophizing [38,39,40,41]. Reducing pain catastrophizing through psychological intervention might be helpful in the pharmacological management of neuropathic pain.

Understanding the molecular mechanisms behind the transmission and modulation of pain, as well as the brain circuitry in relation to sensations and memory, will continue to be a major challenge for the foreseeable future [42,43].

Despite these ongoing efforts, the efficacy of pain treatments in improving patient outcomes remains limited. It is crucial to develop a personalized care and management plan to maintain patients’ activity levels and effectively manage pain [44]. Recently, dietary modification has been considered an integrative lifestyle approach with a positive impact on the common comorbidities of pain, including obesity, type 2 diabetes, cardiovascular mortality, and depression [45,46]. To date, the treatment of pain states has failed to provide adequate relief for many patients. The lack of effective treatment results may also be linked also to our limited understanding of the biological mechanisms underlying this condition. Neuropathic pain management involves pharmacological and non-pharmacological approaches. Considering the possible side effects associated with the use of pharmacological treatments, non-pharmaceutical interventions are advantageous and, therefore, a subject of great interest at present. Non-pharmacological treatments include nutraceuticals, dietary supplements, and natural products with high nutritional and therapeutic value [47,48,49,50].

An individual’s diet has an influence on oxidative stress and inflammation, which are the hypothesized mechanisms contributing to the onset of neuropathic pain. Consumption of an unbalanced diet, rich in refined carbohydrates and saturated fats and low in proteins, promotes the production of reactive oxygen species (ROS) and inflammatory mediators that, eventually, can increase sensitivity to pain [51,52,53]. In particular, carbohydrates are a key source of oxidative stress. Through the oxidation of glucose, superoxide anion radical species are produced and, if not neutralized by antioxidant defense systems, induce the production of ROS [54,55]. In addition, excess carbohydrate promotes lipid peroxidation of low-density lipoprotein (LDL) cholesterol, resulting in further production of free radicals [43].

Additionally, the involvement of the gut in the development of neuropathic pain has been proposed [56,57,58]. There is a close connection between the brain and the gut, known as the “gut–brain axis”, which is mainly regulated by the microbiota. The dysregulation of this axis has been implicated in various pathological conditions, including chronic pain, and may be associated with an imbalanced diet, which can influence the microbes residing in the gut [59,60,61,62]. Alterations in the healthy microbiota can affect levels of immune mediators, metabolites, and neurotransmitters, leading to local immune activation and subsequent systemic inflammation. This systemic inflammation can trigger neuroinflammation, enhancing sensitization of the nervous system and exacerbating neuropathic pain [63,64,65,66].

Despite the existence of all these connections, a direct link between diet and pain remains elusive [67]. Nonetheless, our group, along with numerous other researchers, believes that understanding the mechanisms underlying pain and lifestyle factors remains a significant challenge [68,69,70]. Accumulating evidence has reported the significant role of lifestyle factors—including physical (in)activity, stress, inadequate sleep, and unhealthy diet—in influencing the severity of chronic pain [68,70,71]. These factors exert their effects through both direct mechanisms, such as alterations in neurophysiology, and indirect pathways, such as improvements in mood [68,70]. The critical importance of maintaining an appropriate level of physical activity for individuals with chronic pain is well established [72,73]. Various studies have shown that most individuals with chronic pain experience a reduction in overall physical activity, with the degree of pain severity correlating with lower engagement in physical activity [70,74,75]. Similarly, sleep disturbances, particularly insomnia, are highly prevalent among those suffering from chronic pain, contributing to a bidirectional relationship where sleep disturbances not only exacerbate chronic pain but also correlate with increased depressive symptoms, functional disability, heightened healthcare utilization, and diminished quality of life, especially in adolescents [70,76,77,78].

Meta-analytic findings have further indicated a robust association between overweight, obesity, and chronic pain [70]. In addition to these factors, smoking has emerged as a significant lifestyle determinant in certain individuals with chronic pain. Studies have observed that individuals tend to smoke more frequently when experiencing higher pain levels and make fewer attempts to quit during these periods [70,79,80]. Smoking status in individuals with chronic pain is also associated with an increased risk of alcohol, drug, and opioid dependence, with the combined use of smoking and alcohol further compounding negative effects. Alcohol impairs the body’s ability to metabolize the carcinogenic compounds found in cigarettes and, despite its acute analgesic effect, poses an elevated risk of alcohol abuse in those with chronic pain [70,79].

In this review, we provide a concise overview of pain, highlighting the key markers and biological pathways involved in neuropathic pain. We then explore neuro-nutritional approaches, emphasizing the role of melatonin (MLT) as a potential dietary strategy in the restitution of neuronal redox homeostasis and, thus, in modulating or reducing neuropathic pain. In the present review, the different lines of evidence for the role and mechanism(s) of action of MLT in neuropathic pain states are reported. However, the antinociceptive properties of MLT and MLT agonists and related mechanisms of action remain unclear.

In recent years, the relationship between nutrition and neuropathic pain has gained significant attention, highlighting diet as a potential “simil-therapeutic” intervention. Furthermore, given the role of inflammation and oxidative stress in neuropathic pain, dietary strategies that target these pathways have received increasing attention [43,44,46,49,58,81]. Various dietary strategies and elements have demonstrated efficacy in alleviating neuropathic pain symptoms [71,81,82,83,84,85]; however, further clinical and preclinical studies are needed. Besides MLT, in the present review, we briefly assess the effects of commonly consumed bioactive compounds (such as polyphenols and vitamins) on neuropathic pain. We would like to point out that an increasing amount of data indicates that non-selective compounds directed at more than one molecular target exert promising analgesic effects [86].

We believe that future research on these dietary elements should focus on a detailed understanding of the mechanisms of action of these compounds, as well as in the context of gender differences and in different phases of neuropathic pain. Overall, a detailed understanding of the pharmacokinetic profile and the impact of these compounds on pathophysiological processes may contribute to the development of precise and effective treatments for neuropathic pain, considering the individual condition of patients.

### Neuropathogenic Mechanisms, Gender, and Age as Important Determinants of Pain

As shown in Figure 1, Kankowski et al. (2021) reported some extrinsic factors that summarize the pathogenic mechanisms responsible for pain, which involve the peripherical system, glial, immunocompetent cells, and central pain circuits [24].

Multiple studies have shown that pseudounipolar neurons in the dorsal horn of the spinal cord (Figure 1 top triangle) are the first station of the lateral spinothalamic tract to undergo different molecular processes (outer circle). This mechanism leads to peripheral sensitization, which is hypothesized to be the main driver of neuropathic pain [87,88,89,90,91].

Diabetes mellitus is known to be associated with several health complications, and diabetic peripheral neuropathy is one of the most frequent direct consequences [58,92]. The leading causes of diabetic neuropathy are the metabolic disorders associated with diabetes mellitus. A hyperglycemic state causes a cascade of reactions that end in the accumulation of toxic species, promoting oxidative stress and axonal degeneration, while microvascular changes (which are commonly associated with diabetes mellitus) compromise nerve perfusion, leading to hypoxia and a subsequent loss of nerve function [93,94,95]. Similar to diabetic peripheral neuropathy, chemotherapy treatments are accompanied by a spectrum of health complications. Among others, chemotherapy-induced peripheral neuropathy is a common and debilitating condition associated with certain anti-neoplastic drugs due to significant neurotoxicity resulting from drug-related mechanisms [96,97,98,99,100].

Numerous viruses can also cause neuropathic pain via different mechanisms, depending on the type of pathogen. Through infections, viruses can induce inflammation and injury in the nervous system, the production of cytotoxic substances that increase pain transmission, or an increased immune response that affects organ systems [101,102].

From a molecular point of view, it has also been demonstrated that processes involving voltage-gated ion channels of pseudounipolar neurons may contribute to neuropathic pain. For example, gain-of-function mutations in a particular sodium channel subunit in diabetic patients can cause the hyperexcitability of pseudounipolar neurons, while in hereditary diseases, loss-of-function mutations can lead to insensitivity to pain [24,103].

As described, neuropathic pain is mainly due to pathologic alterations affecting sensitive pathways. However, changes in the descending pathways also appear to participate in the perception of pain. Specifically, the periaqueductal grey matter in the midbrain and the locus coeruleus in the rostral pons are the main descending pathways that contribute to the onset and persistence of neuropathic pain [104,105]. In general, neurons, central nervous system (CNS) glial cells, satellite glial cells, and infiltrating immune cells contribute to the maintenance of pain through the production of cytokines that increase neuronal excitability and promote neuropathic pain [106].

Several studies have also shown a determinant effect of gender/sex on pain [107,108,109,110]. The term “gender” refers to psychosocial dynamics, including societal expectations and their influence on individual behaviors, while the term “sex” reflects the chromosomal complement [110]. Both sex and gender affect health outcomes, leading to differences between men and women in disease risk factors, prevalence, clinical presentation, and response to treatment [110,111]. It has been demonstrated that females may have a lower diffuse pain inhibitory control circuit, inducing neuronal pain [112]. Rivest et al. (2010) showed that male patients with whiplash injury are more sensitive to pain, and, in line with these results, Elklit and Jones (2006) demonstrated that men are more prone to anxiety and disability after this injury [113,114]. One possible explanation is the preference of the emotion-focused coping strategy by males over the symptom-focused approach adopted by females [115]. Gupta et al. (2017) showed that the difference between men and women in pain perception depends on the greater variation in sensorimotor areas of the brain and in the spinal inhibition processes [116]. Interestingly, sex differences occur if the pain persists for many months, evidenced by the fact that mainly T cells are present in the spinal cord after nerve injury in female mice, whereas microglial cells are evident in male mice with the same injury [117]. Due to these data, the therapies for neuroinflammation may need to be adapted to the patient’s sex, hormone status, and comorbidities [3,118,119].

Understanding the relationship between age and pain is a very important challenge. Ruscheweyh et al. (2011) showed that in younger adults, pain is associated with emotional response, while in older subjects, it is mainly associated with the actual pain intensity [120]. In addition, it has been demonstrated that older adults show less sensitivity to cutaneous pain, such as heart pain threshold; however, their sensitivity increases with pain stimuli applied to deeper tissues. This could be related to enhanced pain facilitation combined with decreased pain inhibition [8]. It is known that aging is associated with an increase in pain diseases, and the pathways that need to be explored in detail include the mediators of inflammation, which are responsible for prolonging and amplifying pain [121,122,123].

Given the wide range of causes and the numerous pathological mechanisms involved in neuropathic pain, there is no unified therapy, and several new approaches have been proposed, including dietary interventions [58,124,125].

## 2. Acute Versus Chronic Pain

Acute pain is typically defined as being caused by painful stimuli of limited duration (less than one month), often related to surgical interventions, trauma, and medical procedures [23]. It is widely accepted that certain mediators and factors involved in inducing pain are responsible for the transition from acute to chronic pain (Figure 2) [126].

Chronic pain is a significant global burden, yet its mechanistic basis remains poorly understood. A major challenge lies in identifying the molecular events that allow acute pain, typically resulting from self-resolving tissue injuries, to progress into persistent pain states that outlast the initial injury and can radiate beyond its localized area. While neuroplastic changes are well documented as key contributors, recent studies have emphasized the role of innate immune system activation in driving the transition to chronic pain [127]. Specifically, Fotio et al. (2024) demonstrated that circulating monocytes contribute to pain chronicity through a cell-autonomous mechanism, wherein the suppression of intracellular N-acylethanolamine acid amidase (NAAA)-regulated PPAR-α signaling during the hyperalgesic priming incubation phase plays a critical role [127].

Surgical procedures, such as thoracotomy, mastectomy, and amputation, often result in persistent pain for 50–70% of patients, with 10% experiencing severe pain lasting for at least six months. However, the nature of this pain remains inadequately defined, and a clear boundary between acute and chronic pain is still lacking [126]. Central sensitization is considered the primary mechanism behind chronic pain, with both peripheral and central sensitivity contributing to its development [128,129]. These procedures release inflammatory mediators that initially activate nociceptors. In persistent pain, nociceptors become sensitized, and although this sensitization typically resolves during normal healing, chronic pain triggers alterations in nociceptor behavior. These include changes in gene expression, receptor translocation to the cell membrane, and persistent activation of inflammatory and glial cells. Once these structural alterations occur, chronic pain pathophysiology is established [126]. The regulation of translation appears to be a key mechanism by which nociceptors alter their phenotype, leading to profound hyperexcitability and contributing to the persistence of chronic pain [130].

The physical alterations in neuronal morphology are an important point of pain transmission to the CNS; these changes are defined as neuroplasticity. Neuroplasticity induces the death of the inhibitory interneurons responsible for modulating painful nerve transmission impulses. Furthermore, glial cells amplify acute pain transmission, causing an increase in connections within the CNS and other changes also in higher centers [131,132,133]. Moreover, neuroplasticity can transform acute pain into chronic pain; however, there are several factors that induce pain, such as age, sex, living conditions, and employment status [126,134].

Scientists have suggested that blocking the painful receptors is important for reversing the pathological state. The input responsible for pain is not related to damaged tissue; however, it is tissue-specific, as reported by Voscopoulos and Lema (2010) [126]. At present, it is accepted that there are “sleeping nociceptors” that can be activated after exposure to inflammation and other nociception mediators. These “sleeping nociceptors” can cause an increase in peripheral nociceptive inputs to the CNS [126].

As previously reported, neuropathic pain is a chronic condition resulting from damage to the somatosensory nervous system, significantly impacting quality of life and frequently leading to mood disturbances and sleep disorders. Managing neuropathic pain remains difficult, as many patients fail to achieve sufficient therapeutic relief or tolerate conventional pharmacological treatments [135]. Nutritional interventions, including food intake reduction, caloric restriction, and periods of fasting, have been extensively studied for their potential anti-aging effects in various neurodegenerative disorders [136]. Excessive eating and drinking behaviors have been linked to heightened oxidative stress, which indirectly contributes to the onset and persistence of pain [137].

Caloric restriction not only significantly increases the mechanical threshold (i.e., reducing mechanical hypersensitivity) during periods of reduced caloric intake but also produces long-lasting effects on neuropathic pain throughout the entire testing period [136,138]. This alleviation of hypersensitivity can be, in part, attributed to the potent anti-inflammatory effects associated with caloric restriction [136].

The activation of autophagy in neurons in response to nutrient deprivation has been particularly well documented in hypothalamic neurons, where starvation-induced autophagy plays a key role in regulating food intake and maintaining energy balance. Furthermore, the downstream effects of caloric restriction trigger adaptive responses to energy deficits, enhancing mitochondrial function and subsequently reducing oxidative stress [139,140].

## 3. Mechanisms of Neuropathic Pain Induction

The mechanisms involved in neuropathic pain are intricate and involve a combination of immune response, inflammation, and oxidative stress [141]. Neuronal cells and non-neuronal cells can stimulate neuropathic pain by releasing inflammatory mediators that produce mitochondrial dysfunction and oxidative stress [15]. These cells stimulate neuropathic pain starting from the PNS to the CNS [142]. Peripheral nerve injury induces several changes in the cells reported above in the dorsal root ganglia (DRG), where the cell bodies of primary sensory neurons are found; moreover, these inputs are transferred to the brain, causing pathological amplifications of pain perceptions [143,144,145]. The sex differences and similarities between neuronal and non-neuronal cells are reported in Figure 3. In the PNS and CNS, both sexes have the same mediators promoting pain, while a few of them suppress it [143].

### 3.1. Inflammation and Its Involvement in Pain Transmission

Inflammation is a pathophysiological process associated with pain, inducing the release of several inflammatory proteins [146,147,148,149,150,151,152,153,154].

Cytokines are small proteins that are important mediators for inflammation and pain resolution [155]. Tumor necrosis factor α (TNF-α) is the most important proinflammatory cytokine, even if there are other cytokines in this family, such as interleukin-1β (IL-1β) [156].

#### 3.1.1. A Brief Description of the Most Important Pain Targets in Inflammatory Pathways

As will be indicated, several inflammatory and neurotrophic substances are involved as mediators in neuropathic pain, and they are better explained below [155].

Among these mediators, we first reported the findings for TNF-α as it is upregulated and released from several cells after neuronal injuries compared to other cytokines. TNF-α upregulates other cytokines, inducing the alteration of neuronal components after damage; thus, it is considered a very important cytokine for orchestrating neuropathic pain in the PNS and CNS [146]. TNF-α’s role is mediated by two receptors that are present on the cellular surface, namely, tumor necrosis factor receptor 1 (TNFR1) and tumor necrosis factor receptor 2 (TNFR2). These receptors have different functions related to sex, as described above [157,158], as well as different effects. For instance, TNFR1 triggers neuronal apoptosis, while TNFR2 stimulates vascular pathology without effects on neuronal cells [159]. The different functional mechanisms of these receptors are complex and not yet understood. It seems that TNFR1 cooperates with the nuclear factor-kappa-light-chain-enhancer of activated B (NF-kB) and c-Jun transcription factor in cell death. In the absence of NF-kB activity, TNF-α stimulates apoptosis, whereas elevated NF-kB activity protects against apoptosis [146,160,161,162,163].

Moreover, TNF-α has a strict relationship with matrix metalloproteinases (MMPs), several zinc-dependent endopeptidases comprising collagenase, gelatinase, stromelysin, and other membrane-type MMPs [146]. It is known that TNF-α induces the expression of certain MMPs, including gelatinase, and these proteins control TNF-α expression in several diseases and in neuropathic pain. Ultimately, MMP-mediated effects are thought to occur in relation to changes in the activity of TNF-α and its receptors [146]. In addition, TNF-α has the same functions as IL-1β [164,165].

IL-1β excites both DRG neurons and neurons in the spinal dorsal horn. The action of IL-1β on the primary afferent neurons is critical to the onset and maintenance of pain. As well as its action on microglial cells, IL-1β affects peripheral cells, decreasing K^+^ channel activity and increasing Na^+^ channels. These actions may involve a reduction in the ability to take up glutamate, increasing pain hypersensitivity [155,166,167,168].

We underline the fact that several ILs are involved in neuropathic pain, as described above; however, another interleukin has also been reported. There is strong evidence that nerve injury upregulates both interleukin-18 (IL-18) and interleukin-18 receptor (IL-18R) in astrocytes and microglia. IL-18 blockade modulates injury-induced tactile pain [169,170]. These findings require further study, considering other pain injuries.

##### Chemokines and Nerve Growth Factor

The upregulation of chemokine (C-C motif) ligand 2 (CCL-2) increases pain sensitivity; this has been demonstrated using ccr2 knock-out mice, as they are resistant to the generation of neuropathic pain [171]. CCL-2 is also expressed by microglia and macrophages [172]. Moreover, its presence in some central structures, such as the nucleus accumbens, modulates the generation and transmission of pain [173].

Chemokine (C-C motif) ligand 21 (CCL-21) is present in the vesicles of the DRG axons in the microglia, and it is upregulated and released after nerve injury [174,175,176]. Moreover, it also transmits signals to the astrocytes [177].

There are other chemokines, such as chemokine (C-X-C motif) ligand 1 (CXCL-1) and chemokine (C-X-C motif) ligand 12 (CXCL-12), that have the same effects as the chemokines reported above, and their inhibition attenuates pain [178,179].

Nerve growth factor (NGF) serves as a primary mediator in the development of neuropathic pain; it induces the upregulation of CGRP and substance P in sensory neurons, increasing the sensitivity of nociceptors. These findings have been demonstrated using anti-NGF monoclonal antibodies, which suppress pain in several animal models, including spared nerve injury [180].

### 3.2. Inflammation and Aging

Inflammation mechanisms are more evident in aging subjects; in this period of life, a low level of inflammation is present in the absence of injury (inflammaging), and the mediators of inflammation prolong and amplify the pain [122,123,181,182,183]. Several mediators, such as TNF-α, ILs, inducible nitric oxide (NO) synthase (iNOS), prostaglandin E2 (PGE2), and cyclooxygenase-2 (COX-2), are upregulated during pain, and they are activators of pain receptors during response [122,184,185,186]. Moreover, neuroplasticity-inducing cell death of inhibitory interneurons, as described above, decreases with aging, and this is associated with tissue degeneration [183]. Some aspects of inflammation in young and adult subjects have been reported by Singh et al. (2023) (Figure 4) [183].

### 3.3. Oxidative Stress and Its Involvement in Pain Transmission

Oxidative stress plays an important role in the initiation and maintenance of neuropathic pain, and it is a crucial target for therapeutic strategies [187].

Recently, it has been demonstrated that oxidative stress stimulates the production of reactive oxygen species (ROS), which activate various signaling pathways, including those involved in the transmission and modulation of pain from the PNS to the CNS.

ROS trigger mitochondrial dysfunction, and vice versa; therefore, the pathways contribute to the development and maintenance of pain, inducing alterations in the disruption of cellular physiology [141,188]. Growing evidence shows that increased ROS/reactive nitrogen species (RNS) levels caused by several clinical conditions may have negative effects on lipids, proteins, nucleic acids, organelles, and antioxidant defenses, exacerbating the responses [15].

Multiple stress pathways and several targets have been demonstrated to be involved in oxidative stress during pain transmission [187,189,190]. It is important to remember that oxidative stress is also closely linked to inflammatory mechanisms [14,15]; therefore, the targets involved in oxidative stress and inflammation could be better evaluated for managing pain and focalizing new therapies.

#### 3.3.1. A Brief Description of the Most Important Pain Targets in Oxidative Stress Pathways for Neuropathic Pain

Various stress pathways and mediators of oxidative stress participate in the pathogenesis of neuropathic pain, and some of them are reported below [14,15,191,192,193].

##### Transient Receptor Potential Channels

Calcium channels from the transient receptor potential (TRP) superfamily, which includes several members, have recently been shown to play an important role in the development of pain mediated by sensory neurons [15]. However, the way in which these channels regulate pain signaling is relatively unexplored. We summarized the relationship existing between oxidative stress and TRP channels to evaluate possible therapeutic antioxidant therapies.

The effects of ROS and RNS on neuronal cells in neuropathic pain and the involvement of TRP channels have been thoroughly reported by Carrasco et al. (2018), and we provide a summary in Figure 5 [15].

In this regard, increased levels of calcium ions (Ca^2+^) seem to play an important role in the physiology of neuropathic pain; Ca^2+^ enters cells through cation channels and, among them, the TRP family. The TRP family is divided into 30 channels within seven subfamilies; in particular, nine members of the TRP subfamily are activated by oxidative stress, and they are associated with the transient receptor potential melastatin (TRPM) family [194]. Their expression is high in the DRG and trigeminal ganglia neurons during neuropathic pain; they play an important role in pain mediated by sensory neurons and the DRG [15].

One member of the TRPM family, TRPM4, requires more extensive research; it is a non-selective Ca^2+^ channel that is activated by an increased intracellular adenosine triphosphate (ATP) concentration and oxidative stress [195]. Moreover, an increased expression of TRPM4 has been observed during oxidative stress without an altered ATP concentration; in this case, apoptosis and necrosis were involved [195]. Other mechanisms may also be involved in the pathophysiological pathways triggered by oxidative stress in neuropathic pain. One of these is considered another marker, aquaporin-1; this protein has been found to increase significantly for up to 11 months in neuronal and non-neuronal cells after PNS injury. Experimental findings suggest that the administration of an antioxidant, MLT, not only reduces protein levels but also has positive effects on the dorsal horn of the spinal cord [196,197]. This concept is better considered in the second part of the review, in which we describe the role of this indolamine as a neuro-nutritional approach for neuropathic pain.

##### Nuclear Factor Erythroid 2-Related Factor

Recently, the nuclear factor erythroid 2-related factor (Nrf2) became important in managing neuropathic pain [187,198]. The increased expression of Nrf2 modulates oxidative stress, reducing the hyperstimulation of neurons that often leads to pain [199]; Nrf2 then maintains the mitochondrial cytoarchitecture, affecting cellular redox homeostasis [200]. It has been demonstrated that Nrf2 regulates inflammation by increasing the expression of anti-inflammatory mediators, thus regulating the cellular environment. Its activation determines the survival of cells during stressful conditions, regulating the genes vital for metabolism and anti-inflammation pathways. Under physiological conditions, Nrf2 is present in the cytoplasm bound to a Kelch-like ECH-associated protein (Keap1), which is important for maintaining cellular health [201,202,203,204]. Upon oxidative stress, Nrf2 translocates into the nucleus and binds to the antioxidant response element (ARE) in the promoter regions of target genes. ARE is a sequence of genes that stimulates the production of several proteins with antioxidant and anti-inflammatory mechanisms (Figure 6) [205,206].

Thus, the activation of Nrf2 pathways upregulates the expression of antioxidant and cytoprotective genes, as shown in Figure 7.

There are important considerations regarding the link between mitochondria, Nrf2 expression, and oxidative stress.

Mitochondria are crucial for maintaining intracellular calcium levels, and the cooperation between these organelles and ROS results in cellular survival [207,208].

Ultrastructural alterations in mitochondria have been found mainly in the PNS [188], and Nrf2 is one of the main regulators of cellular health, checking structural integrity and protection from oxidative stress (Figure 8) [209].

All of these effects suggest that Nrf2 is a promising marker of reduced inflammation and oxidative stress in various pain conditions in both the CNS and the PNS [187,210,211,212,213]. Clinical trials could be conducted to evaluate the anti-inflammatory and antioxidative stress of Nrf2 using antioxidant substances in patients with neuropathic pain [214]. Considering the relationship between pain, inflammation, and oxidative stress, the connection will be successively described.

## 4. Nutrition and Neuropathic Pain

Several studies have shown a relationship between nutrition and neuropathic pain [43,45,50]; however, it is unclear how nutritional factors could interact with pain and the pathways that determine this relationship. The identification of these mechanisms can be useful for nutritional evaluations and treatments in pain management [45]. The new proposed interactions between pain and nutrition are reported in Figure 9.

An altered intake of specific nutrients is linked to oxidative stress, cell death, and tissue damage, all of which can activate toll-like receptors (TLRs). These receptors, through cascading reactions, induce pro-inflammatory events, among which is glial cell activation. The dysregulated activation of glial cells via various mechanisms eventually causes heightened sensitization of the nervous system [106,215,216,217,218]. Based on these considerations, the intake of antioxidant and anti-inflammatory foods can be a strategy to alleviate pain [81,219,220]. As reported by Curatolo and Moavero (2021) [48], neuropathic pain management involves pharmacological and non-pharmacological approaches. Considering the possible side effects associated with the use of pharmacological treatments, non-pharmaceutical interventions are advantageous and, therefore, a subject of great interest at present. Non-pharmacological treatments include nutraceuticals, dietary supplements, and natural products with high nutritional and therapeutic values [47,48,49,50].

Bioactive compounds derived from plants have garnered significant interest as potential agents for neuropathic pain management [221,222]. These compounds, which include flavonoids, terpenoids, alkaloids, and phenolic acids, exhibit a range of pharmacological properties, including antioxidant, anti-inflammatory, and neuroprotective effects [223]. Notably, several phytochemicals can modulate pain pathways by influencing mechanisms, including oxidative stress, inflammation, and ion channel activity, all of which play a role in neuropathic pain pathophysiology [222,224]. While phytochemicals are not yet a definitive solution, they represent a significant advancement in neuropathic pain management and hold promise as complementary or alternative therapies. Furthermore, MLT could potentially be added to the classical class of drugs with its peripheral (anti-inflammatory) and central (activation of the endogenous opioid system and benzodiazepine–GABAergic pathway) actions [225]. Furthermore, in humans, various factors, including ethnicity, gender, genetics, lifestyles, and types of neuropathic pain, also need to be taken into account when designing clinical trials. Personalized nutrition with bioactive compounds should also be explored in future human trials.

**Figure 10:**
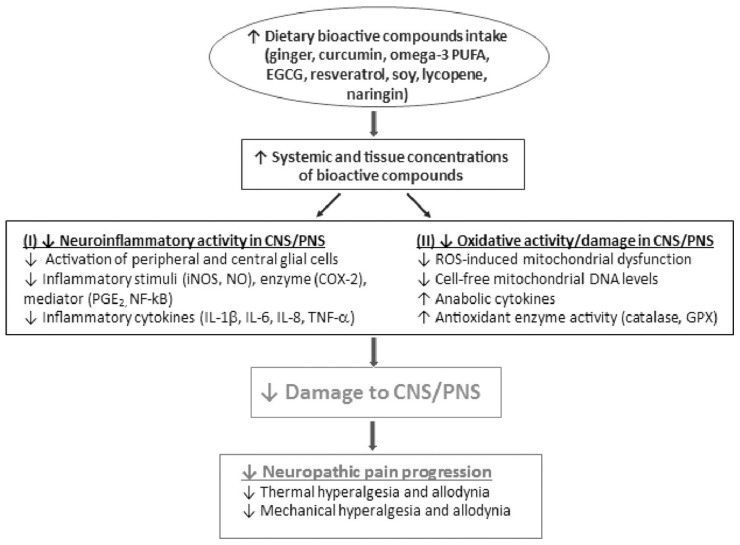
Bioactive compounds in the management of neuropathic pain. Neuropathic pain is associated with an increase in neuroinflammation and oxidative stress in both the central nervous system and peripheral nervous system. Increased intake of dietary bioactive compounds would increase the concentrations of bioactive compounds in circulation and tissues, leading to a reduction in neuroinflammation activity and oxidative activity/damage in both CNS and PNS. The consequence is the mitigation of the progression of neuropathic pain. CNS, central nervous system; PNS, peripheral nervous system; COX-2, cyclooxygenase-2; EGCG, epigallocatechin gallate; GPX, glutathione peroxidase; IL-1β/6/8, interleukin-1β/6/8; TNF-α, tumor necrosis factor α; iNOS, inducible nitric oxide synthase; NF-kB, nuclear factor-kappa-light-chain-enhancer of activated B cells; NO, nitric oxide; PGE2, prostaglandin E2; ROS, reactive oxygen species. The arrow pointing up indicates “increase”; whereas the arrow pointing down indicates “decrease”. Illustration from Shen et al. [236]. (Licensed number 6000670861939).

**Figure 1:**
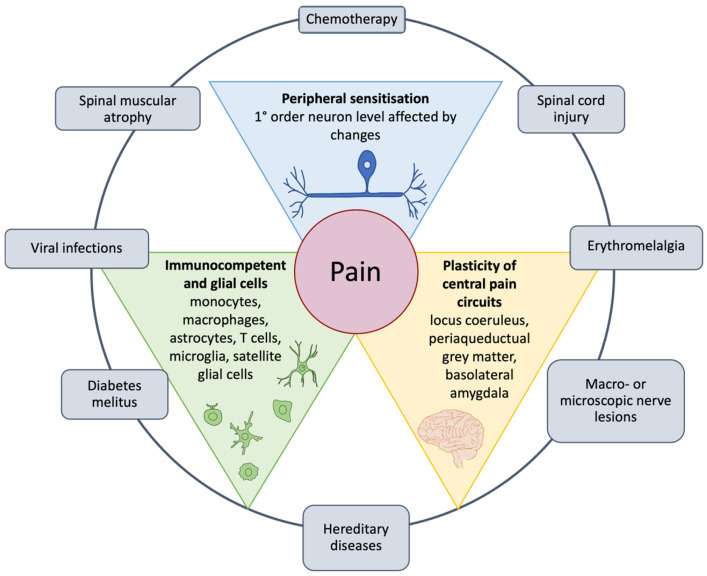
The main pathogenic mechanisms, such as infections, muscular atrophy, several lesions, erythromelalgia, hereditary diseases, and diabetes mellitus (outer circle), and the central structures involved in pain processing (internal triangles). Illustration adapted from Kankowski et al. [24].

**Figure 2:**
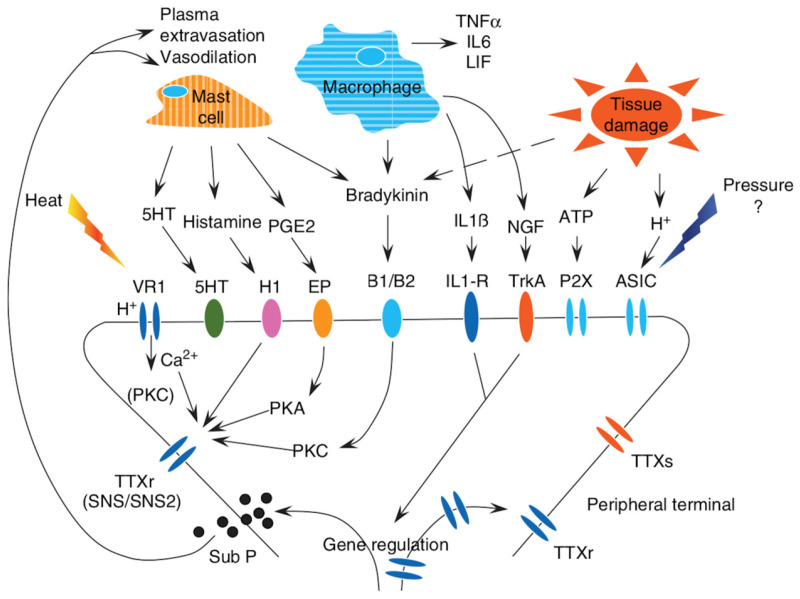
Peripheral mechanisms of nerve fiber sensitization. Peripheral neurons have numerous ligand and voltage-gated ion channels, many of which use the PKA and PKC signaling pathways. PKA, protein kinase A; PKC, protein kinase C; TNF-α, tumor necrosis factor α; IL-6, interleukin-6; LIF, leukemia inhibitory factor; 5HT, 5-hydroxytryptamine; PGE2, prostaglandin E2; IL-1β, interleukin-1β; NGF, nerve growth factor; ATP, adenosine triphosphate; H^+^, hydrogen ion; VR1, vanilloid type 1 receptor; H1, histamine type 1; EP, prostanoid receptor EP subtype; B1/B2, bradykinin receptors; IL1-R, interleukin-1 receptor; TrkA, tyrosine kinase A; P2X, purinergic receptor subtype P2X; ASIC, acid-sensing channel; Ca^2+^, calcium; TTXr (SNS/SNS2), protein tetrodoxin-resistant voltage-gated sodium channel; TTXs, tetrodoxin-sensitive voltage-gated sodium channel; Sub P, substance P. Illustration from Voscopoulos and Lema [126] (license number 5981260617219).

**Figure 3:**
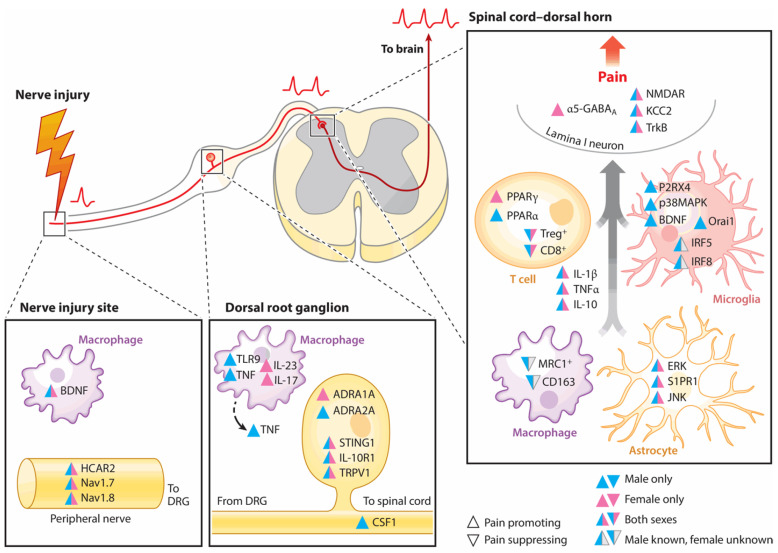
Sex differences and similarities in the key cellular and molecular factors involved in neuropathic pain. Injured peripheral nerves send signals to the dorsal horn of the spinal cord, which then transmits them to the brain. Blue represents male-only involvement, pink represents female only, blue and pink together represent sex similarities, and blue with gray represents studies that were tested in male rodents only. Triangles pointing up indicate mediators that promote pain, whereas triangles pointing down indicate that mediators suppress pain effects. α5-GABA_A_, γ-aminobutyric acid A receptor, alpha 5; ADRA1A, adrenoceptor alpha 1A; ADRA2A, adrenoceptor alpha 2A; BDNF, brain-derived neurotrophic factor; CD163, macrophage-associated antigen; CD8^+^, T cell expressing CD8; DRG, dorsal root ganglia; ERK, extracellular signal-regulated kinase 2; IL-10, interleukin-10; IL-10R1, interleukin-10 receptor; IL-17, interleukin-17; IL-1β, interleukin-1β; IL-23, interleukin-23; IRF5, interferon regulatory factor 5; IRF8, interferon regulatory factor 8; JNK, JUN N-terminal kinase; KCC2, neuronal K-Cl cotransporter; MRC1^+^, macrophage mannose receptor 1-like protein 1; Nav1.7, voltage-gated sodium channel 1.7; Nav1.8, voltage-gated sodium channel 1.8; NMDAR, N-methyl-D-aspartate receptor; Orai1, calcium release-activated calcium channel protein 1; P2RX4, purinergic receptor P2X 4; p38MAPK, p38 mitogen-activated protein kinase; PPARα, peroxisome proliferator-activated receptor alpha; PPARγ, peroxisome proliferator-activated receptor γ; S1PR1, sphingosine-1-phosphate receptor 1; STING1, stimulator of interferon response CGAMP interactor 1; TLR9, Toll-like receptor 9; TNF, tumor necrosis factor; Treg^+^, regulatory T cell; TrkB, neurotrophic receptor tyrosine kinase 2; TRPV1, transient receptor potential cation channel subfamily V member 1. Illustration from Ghazisaedi et al. [143]. This is an open-access article distributed under the terms of the Creative Commons Attribution 4.0 license.

**Figure 4:**
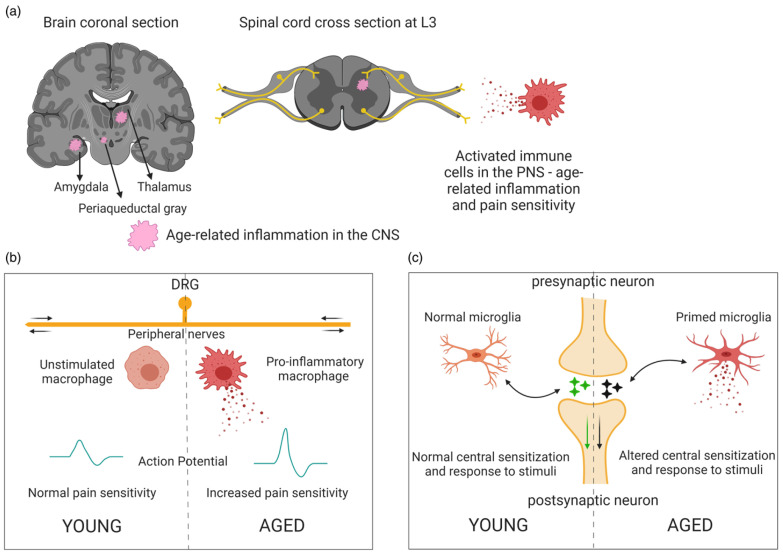
Some aspects of pain in aging: (**a**) Chronic low-grade inflammation in aging is characterized by activated microglia in the CNS and activated macrophages in the PNS. (**b**) Differences in PNS inflammatory state, neuronal activity and pain response in aged peripheral tissues compared to young. Chronic secretion of pro-inflammatory mediators damages nerves and alters pain sensitivity in aged peripheral tissues. (**c**) CNS immune sensitisation or microglia become "primed", leading to behavioural deficits in the elderly. CNS, central nervous system; PNS, peripheral nervous system; DRG, dorsal root ganglia. Illustration from Singh et al. [183]. This is an open-access article distributed under the terms of the Creative Commons Attribution 4.0 license.

**Figure 5:**
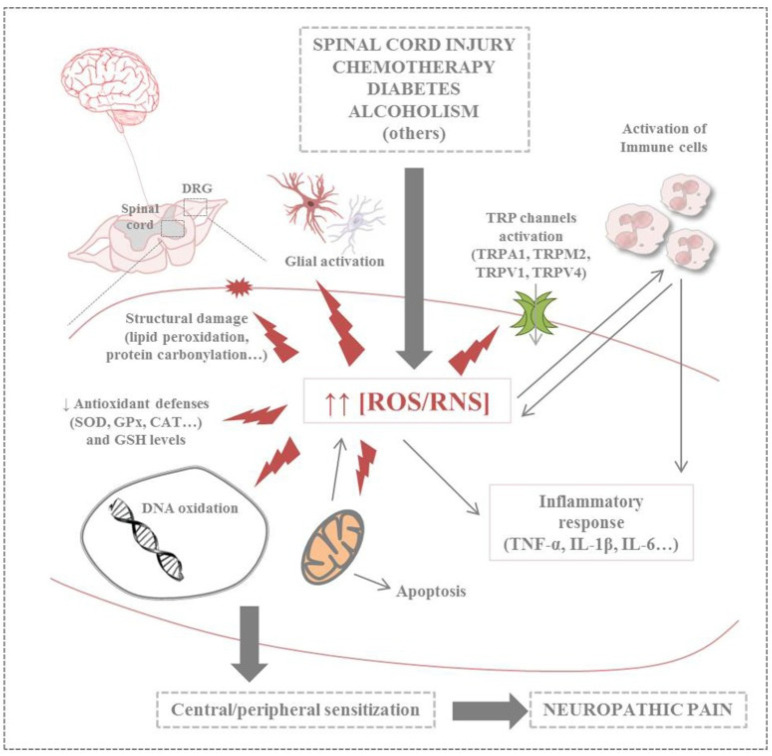
The effects of nitro-reactive stress and reactive oxygen species on neuronal cells in neuropathic pain. DRG, dorsal root ganglia; SOD, superoxide dismutase; GPx, glutathione peroxidase; CAT, catalase; GSH, glutathione; ROS/RNS, reactive oxygen species/reactive nitrogen species; TRP, transient receptor potential; TRPA1, transient receptor potential ankyrin 1; TRPM2, transient receptor potential melastatin 2; TRPV1/4, transient receptor potential vanilloid 1/4; TNF-α, tumor necrosis factor α; IL-1β/6, interleukin-1β/6. Illustration from Carrasco et al. [15]. This is an open-access article distributed under the terms of the Creative Commons CC-BY license.

**Figure 6:**
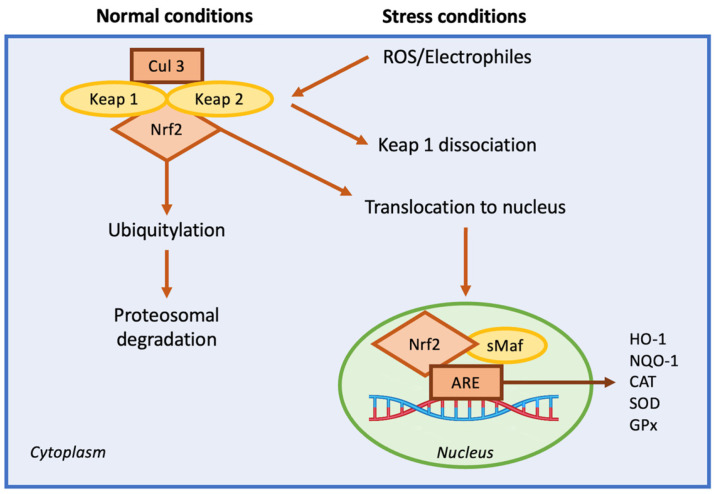
Nrf2’s role under physiological and stressful conditions. Keap 1/2, Kelch-like ECH-associated protein 1/2; Cul 3, Cullin 3; Nrf2, nuclear factor erythroid 2-related factor; ARE, antioxidant response element; sMaf, small Maf protein; NQO-1, NAD(P)H quinone oxidoreductase 1; HO-1, heme-oxygenase-1; CAT, catalase; SOD, superoxide dismutase; GPx, glutathione peroxidase. Illustration adapted from Petrikonis et al. [141].

**Figure 7:**
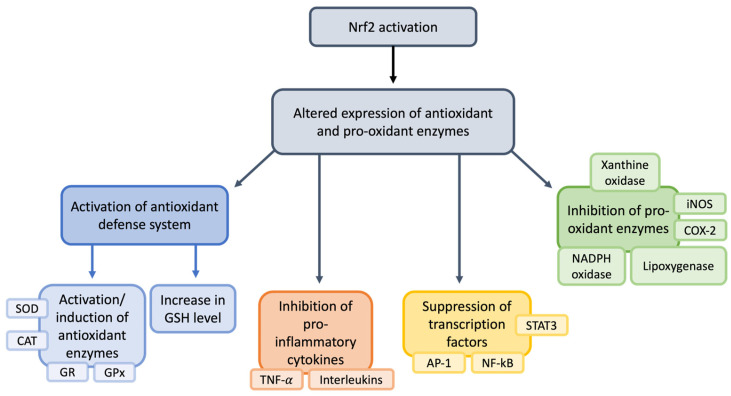
Antioxidant effects following Nrf2 activation in cells. Nrf2, nuclear factor erythroid 2-related factor; TNF -α, tumor necrosis factor α; AP-1, activator protein 1; NF-κB, nuclear factor-kappa-light-chain-enhancer of activated B cells; STAT3, signal transducer and activator 3; COX-2, cyclooxygenase-2; iNOS, inducible nitric oxide synthase; SOD, superoxide dismutase; CAT, catalase; GPx, glutathione peroxidase; GR, glutathione reductase; GSH, glutathione. Illustration adapted from Petrikonis et al. [141].

**Figure 8:**
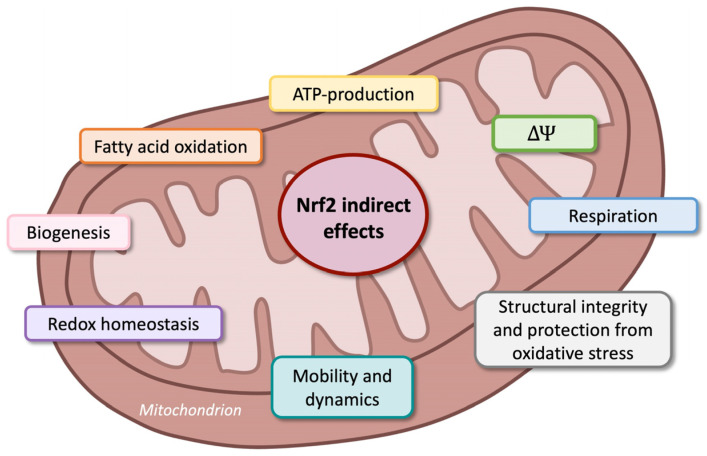
Nrf2 and its indirect actions on mitochondrial functions. ATP, adenosine triphosphate; ΔΨ, potential of the mitochondrial membrane. Illustration adapted from Petrikonis et al. [141].

**Figure 9:**
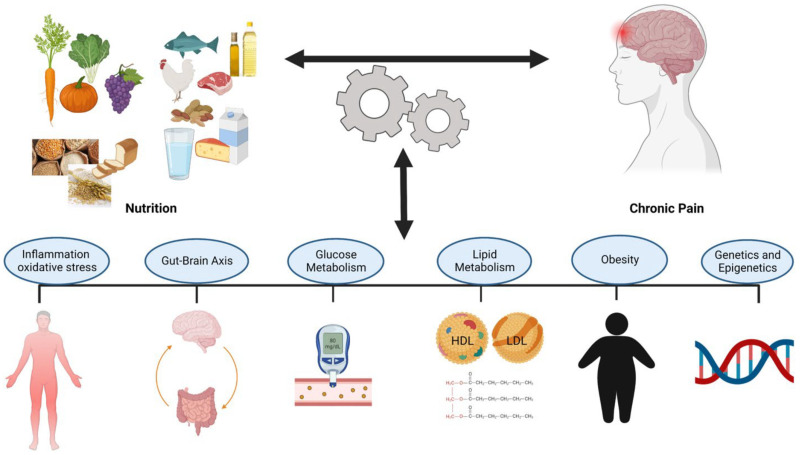
The interactions between chronic pain and nutrition. HDL, high-density lipoprotein; LDL, low-density lipoprotein. Illustration from Elma et al. [45]. This article is an open-access article distributed under the terms and conditions of the Creative Commons Attribution CC BY license.

As reported above, neuropathic pain is thought to be the result of inflammation and oxidative stress, and clinical studies have demonstrated links between these conditions and diet [43]. Allison et al. (2016) conducted a randomized, parallel-group, controlled clinical trial involving 20 patients with varying levels and severities of spinal cord injury (central neuropathic pain) [226]. The participants were assigned either to a 12-week anti-inflammatory diet group (twelve participants) or to a control group (eight participants). The anti-inflammatory diet intervention focused on the elimination of common food intolerances and inflammation-inducing foods, as well as the introduction of foods and supplements with established anti-inflammatory properties. Foods removed from the diet included those with high glycemic indices (such as refined wheat products and refined sugars), common intolerances such as cow’s milk, and foods that negatively influence cardiovascular health, such as hydrogenated oils. Participants also consumed daily supplements with established anti-inflammatory benefits. Omega-3 was taken in soft gel form, whereas chlorella, curcumin, and antioxidants were taken in pill form, containing 100 mg of coenzyme Q10, 200 mg of n-acetylcysteine, 150 mg of mixed tocopherols, 100 mg of DL alpha lipoic acid, 60 mg of green tea extract, 5.5 mg of zinc, and 100 μg of selenium at a dosage of two per day. A vegetable-based protein powder containing 27 g of protein was taken at a dosage of one scoop each morning. Participants were asked to complete the Neuropathic Pain Questionnaire at each of the three testing sessions (baseline, one month, and three months of diet treatment) as a means of assessing self-reported neuropathic pain. Serum inflammatory markers were also evaluated in all participants. The study demonstrated a significant association between diet-induced reductions in inflammation and improvements in neuropathic pain scores [226]. This finding suggests that dietary modifications may directly target the underlying mechanisms of neuropathic pain, explaining their effectiveness as a treatment modality (Figure 10). Moreover, dietary interventions provide some advantages over traditional pharmacological treatments, which primarily focus on symptom relief through downstream targets, such as the direct reduction in neuronal hyperexcitability [226].

Schell et al. (2017) evaluated the effects of dietary strawberries on pain, markers of inflammation, and quality of life indicators in obese adults with knee osteoarthritis (peripheral pain) [227]. The patients were randomly assigned to one of the two study groups in a 26-week crossover study: those on the strawberry diet and those on the control diet. During the active treatment phase, the participants consumed 50 g of freeze-dried strawberry powder reconstituted in water twice a day for 12 weeks (this dose of strawberry powder is equivalent to approximately 500 g of fresh strawberries). The participants were also instructed to refrain from consuming other berry products during the study and to maintain their usual diet and physical activity. Knee pain scores were assessed using the Visual Analog Scale for Pain, Measures of Intermittent and Constant Osteoarthritis Pain (ICOAP), and Health Assessment Questionnaire—Disability Index (HAQ-DI) questionnaires, which were completed at baseline and at weeks 12, 14, and 26 of the study. The HAQ-DI was used to assess functional ability, comprising 20 items distributed across eight dimensions (dressing, arising, eating, walking, reach, grip, hygiene, and daily activity). Notably, strawberry supplementation led to significant decreases in constant, intermittent, and total knee pain scores and an improved disability index [227].

Cirillo et al. (2023) [85] evaluated the impact of dietary changes based on the Mediterranean diet on pain perception in 35 women with endometriosis (peripheral neuropathic pain), exploring its relationship with oxidative stress. Patients were asked to indicate the perceived pain intensity using the Visual Analogue Scale (VAS) [85]. The Mediterranean diet consists of 50–55% carbohydrates, 25–30% total fat (≤10% saturated), and 15–20% protein. The authors observed a clear trend linking pain relief (including non-menstrual pelvic chronic pain, dyspareunia, dysuria, and dyschezia) to adherence to Mediterranean dietary patterns. The Mediterranean diet may alleviate endometriosis-related pain through various mechanisms, including its anti-inflammatory and antioxidant effects. In particular, fish rich in omega-3 fatty acids and extra virgin olive oil were recommended due to their anti-inflammatory properties. In addition to their anti-inflammatory effect, the components of the Mediterranean diet also exert an eupeptic effect [85].

Moreover, the benefits of nutrients and specific diets for conditions like anxiety, depression, cognitive decline, and neurodevelopmental disorders, which frequently accompany chronic pain, have been evaluated [228]. It is known that poor diet plays an important role in the onset, prognosis, and maintenance of neuropathic pain [74,229,230,231,232,233].

The decreased antioxidative and detoxifying ability of the body plays a role in inflammation pain pathways [43]; to the contrary, increased dietary antioxidant intake and increased detoxifying ability of the body alleviate nociceptive and neuropathic pain in the population [227,234]. Thus, it is important to adopt a multidisciplinary approach to identify nutrition-related risk factors associated with pain and to provide nutrition-based treatment strategies. Nutritional neuroscience is a recently developed science that evaluates the effects of nutrients on the CNS and PNS [235].

### 4.1. Melatonin: Secretion and Effects

In mammals, MLT (N-acetyl-5-methoxytryptamine) is an indoleamine secreted mainly by the pineal gland during darkness, but it is also synthesized in other organs such as the gut [237,238,239,240]. Due to its small size and amphiphilic property, which makes it soluble in both water and lipids, MLT can enter all cellular components. It also has the advantage of being safe and nontoxic. Its administration in several clinical trials in both adults and children showed no adverse effects or only mild side effects, such as dizziness, drowsiness, headaches, and nausea [47,241,242,243,244].

MLT has the function of managing the mammalian circadian rhythm [78,245]. In fact, suppression in the secretion of MLT has been observed in shift workers exposed to artificial lighting at night, as well as in individuals who use light-emitting electronic devices, such as smartphones or tablets, before bedtime [77,246]. In addition to its important role in regulating the sleep–wake cycle, MLT is implicated in many other biological functions through its antioxidant, anti-inflammatory, antinociceptive, anti-apoptotic, and analgesic properties [77,247,248,249,250,251,252,253,254,255]. Its antioxidant and free radical scavenger properties are exerted by directly eliminating hydroxyl radicals, hydrogen peroxide, and other oxygen radicals. It also regulates the activity and expression of antioxidant enzymes, such as glutathione peroxidase and superoxide dismutase, and strengthens immunological functioning [256,257,258,259,260,261].

MLT can also act as an antinociceptive, anxiolytic, and antidepressant [262,263,264,265,266]. As reported by Mauriz et al. (2013), MLT can downregulate inflammatory mediators, including prostaglandins and cytokines, which are markers known for their pain-sensitizing actions [267]. MLT inhibits COX-2, one of the enzymes responsible for the inflammatory response, and increases the analgesic effects of nonsteroidal anti-inflammatory drugs (NSAIDs), such as ibuprofen [268]. In addition, this multitasking indoleamine may also interact with other painkillers with adjuvant/beneficial effects [266,269,270].

The numerous effects of MLT may be dependent on or independent of binding to specific receptors [271]. MT1 and MT2 are two membrane-bound G-protein-coupled receptors (GPCRs) that bind MLT with high affinity. They are widely expressed throughout the CNS, the cardiovascular system, and many other peripheral regions, with a tissue-specific distribution [266,272,273]. By interacting with these regions, MLT causes a decrease in cyclic AMP levels and a consequent reduction in nociception [241]. MT2 receptors play an important role in the modulation of pain mechanisms, having analgesic effects [274,275,276,277,278,279]. In a model of median nerve demyelination induced by lysophosphatidylcholine, MLT administration reduces the release of pro-inflammatory cytokines from glia. It also induces MT2 receptor-mediated suppression of glial intracellular Ca^2+^ levels and inhibition of microglial p38 mitogen-activated protein kinase, preventing the development of neuropathic pain behavior [280,281]. Although MT1 receptors are involved in the modulation of pain, their effects are less significant than those of MT2 receptors. Instead, MT1 receptors are important in the regulation of the circadian rhythm and several physiological processes [282,283,284,285]. As demonstrated by several studies, sleep disorders may increase the risk of developing neuropathic pain and anxiety-like behavior, and total sleep deprivation increases sensitivity to the pain stimulus and lowers the pain threshold [76,284,286,287,288]. Wang et al. (2021) demonstrated that MLT supplementation in a mouse model of acute sleep deprivation through the NF-kB pathway can ameliorate anxiety-like behavior [289]. Sleep disorders are inevitable in most pain conditions, and this induces an alteration in the circadian rhythm [253]. It seems that normalizing the levels of MLT secretion and the circadian rhythm improves sleep disorders [290]. Moreover, it seems that MLT can interact with opioid, adrenergic, and cannabinoid receptors, which are involved in pain regulation and can mediate MLT modulation of the pain signaling pathway [77,256,267,291,292]. It is important to remember that this relationship is not completely understood; however, several studies have reported this involvement [293].

**Figure 11:**
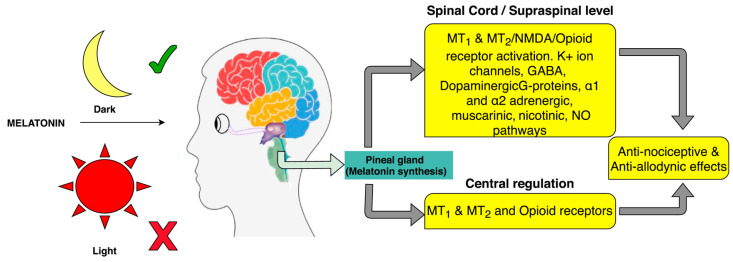
Mechanisms of action of melatonin through which it promotes anti-allodynic and antinociceptive effects and regulates its synthesis through the pineal gland in light–dark cycles. Melatonin promotes antinociceptive and anti-allodynic effects not only through regulation of MT1/MT2 receptors in the spinal cord and brain but also by interacting with other receptors such as the GABAergic system, the nitric oxide (NO)–arginine pathway, the N-methyl-D-aspartate (NMDA) system, and the dopaminergic system. GABA, γ-aminobutyric acid; NMDA, N-methyl-d-aspartate; NO, nitric oxide. Illustration from Kuthati et al. [262] (license number 5943680387314).

In addition to MT1 and MT2 receptors, MLT interacts with other receptors, such as γ-aminobutyric acid type A (GABA_A_) receptors and the NO–arginine pathway (Figure 11) [294,295,296].

In addition to its antinociceptive effects, MLT has a free radical scavenger action on free radicals such as NO, singlet oxygen, and ROS/RNS in the DRG [297,298].

The first study on the effectiveness of MLT on pain was published in 1969, showing that, during darkness, when there is an increase in plasma MLT levels, mice are less sensitive to nociception [299]. MLT is considered effective and safe in the management of neuropathic pain, as shown in several animal models [262,300,301] and as reported by Curatolo and Moavero (2021) [48].

#### 4.1.1. Analgesic Effect of Melatonin in Human Studies

In humans, the analgesic effect of MLT on neuropathic pain has not been particularly studied and evaluated (Table 1). The present review indicates that MLT exhibits several mechanisms that can contribute to antinociception. Therefore, the potential clinical value of MLT is not negligible. However, as with all new drugs, a significant number of studies must be performed to provide evidence for their benefits in the clinic.

To the best of our knowledge, MLT has been investigated in fibromyalgia (FM), irritable bowel syndrome (IBS), and migraines. FM is a musculoskeletal pain syndrome; IBS is a painful condition of the gut; migraines are a common condition characterized by attacks of severe headaches [302].

Few clinical trials have examined the role of MLT in treating neuropathic pain. In the following paragraph, we will describe the main effects of MLT in patients with FM, IBS, or migraine.

It has been demonstrated that patients with FM have low levels of MLT precursors (L-tryptophan and serotonin), and these findings support the importance of this indolamine in the management of chronic pain [303]. To the best of our knowledge, the administration of MLT to FM patients has been carried out in very few studies. Citera et al. (2000) [304] performed a pilot open-label study involving 21 female FM patients who orally received 3 mg of MLT for four weeks (30 min before bedtime). The authors reported that MLT therapy reduced pain, fatigue, and depressive symptoms [304,305]. Thereafter, Hussain et al. (2021) performed a double-blind placebo-controlled study involving 101 FM patients who received different MLT doses (3 or 5 mg/day), and MLT was administered alone or in combination with fluoxetine [306]. Fluoxetine was given daily in the morning, and MLT was given each evening for eight weeks. The authors showed that administration of both MLT alone and fluoxetine alone significantly improved pain, fatigue, depression, and morning stiffness. Notably, MLT alone also improved also sleep/rest activity. The combination of fluoxetine and MLT promoted even greater reductions in both anxiety and depressive symptoms, with a reduction in fatigue symptoms in addition to each of the symptoms improved by each drug administered individually. The authors concluded that MLT may be an alternative to conventional drug therapy, and it is also a safe treatment [306]. Acuna-Castroviejo et al. (2006) performed a study in which 6 mg/day of MLT was given orally (60 min before the expected sleeping time) to four FM patients who also received other medication, including chronic analgesics, antidepressants, sedative hypnotics, and—in one case—opioids [307]. After 15 days of MLT treatment, all patients experienced significant improvements in their sleep/wake cycle and, notably, a significant reduction in pain severity and fatigue. These outcomes improved, in turn, the behavioral symptoms, including depression [307].

To date, two randomized placebo-controlled clinical trials have administered MLT against IBS, a functional gastrointestinal disorder distinguished by recurring abdominal pain and alterations in bowel frequency and habit. Song et al. (2005) performed a clinical trial involving 40 patients in which 3 mg/day of MLT was administered for two weeks [308]. The MLT-treated group reported abdominal and rectal pain reductions [308]. Lu et al. (2005) [309] included 24 IBS patients who were randomized to receive either 3 mg of MLT at night or a placebo for eight weeks, followed by a four-week washout period. Afterward, the patients received the reverse treatment (placebo or MLT) for another eight weeks [309]. Notably, the MLT-treated IBS patients reported a significantly greater pain reduction and improvements in their IBS symptoms compared to the placebo group [309].

In a study involving 146 migraine sufferers with additional pain syndromes, significantly lower urinary 6-sulphatoxy-melatonin was observed with respect to controls or migraine patients without additional pain syndromes [310]. Claustrat et al. (1997) performed a clinical trial that involved six migraine patients and nine healthy controls [311]. The MLT-treated group received a nocturnal 20 µg infusion to evoke plasma MLT levels, which are slightly higher than the physiological secretion peak. The migraine patients reported significant pain relief and absence of side effects [311]. Moreover, Tabeeva et al. (2011), in a pilot clinical trial, enrolled 20 migraine patients who received 25 mg/day of agomelatine (melatoninergic antidepressants) for three months [312]. Agomelatine treatment decreased both the frequency and duration of migraine attacks and thus reduced the intensity of pain in migraine patients. The reduction in depression severity and normalization of night sleep were also observed in migraine patients treated with agomelatine [312].

Peres et al. (2004) performed a trial including patients with migraines; they received 3 mg as a prophylaxis 30 min before bedtime and showed a reduction in headache frequency, intensity, and duration [313]. Another study showed that the prolonged release of MLT (2 mg one hour before bedtime) had no positive effects on migraines; these results may be related to the fact that the beneficial actions of MLT may be dose-dependent [314].

Recently, Mehramiri et al. (2024) [263] showed that 3 mg of MLT one hour before bedtime for two months reduced migraine frequency and attacks in a clinical trial; these beneficial effects were present until the fourth month after treatment. Moreover, scientists have suggested that MLT has an antinociceptive nature due to its action on opioid, γ-aminobutyric acid (GABA), adrenergic, and serotonergic receptors, as well as MT receptors [245,263,292].

All of these studies found no side effects; the high safety profile represents a possible and interesting analgesic or add-on to pharmacological treatments.

**Table 1:** Significant studies on melatonin and pain.

Study	Pain Model	Study Design	Participants	Outcomes
Jallouli et al., 2025 [253]	Multiple sclerosis	Randomized controlled trial	27 patients with multiple sclerosis	Improvements in dynamic postural stability and walking performance
Mehramiri et al., 2024 [263]	Migraines	Double-blind, randomized clinical trial	60 patients with episodic migraines	Reduction of frequency and duration of migraine attacks
Alstadhaug et al., 2010 [314]	Migraines	Randomized, double-blind, placebo-controlled crossover study	Men and women, aged 18–65 years, with migraines but otherwise healthy, experiencing 2–7 attacks per month	No reduction in attack frequency and no improvement in sleep quality
Acuna-Castroviejo et al., 2006 [307]	Fibromyalgia	Open study	4 patients who also received other medication, including chronic analgesics, antidepressants, sedative hypnotics, and in one case opioids	Improvements in the sleep/wake cycle and significant reduction of pain severity and fatigue
Lu et al., 2005 [309]	Irritable bowel syndrome	Double-blind placebo-controlled study	IBS patients (aged 20–64 years; 24 female) with sleep disturbances	Significant improvements in mean IBS scores after treatment with melatonin (3.9 ± 2.6) than with a placebo
Song et al., 2005 [308]	Irritable bowel syndrome	Randomized, double-blind, placebo-controlled study	17 female patients satisfying the Rome II criteria for IBS	Decreased mean abdominal pain score and increased mean rectal pain threshold
Peres et al., 2004 [313]	Migraines	Open-label trial	40 patients with episodic migraines with or without aura	Decreased headache frequency, headache intensity on a 0 to 10 scale, and duration in hours
Citera et al., 2000 [304]	Fibromyalgia	Open-label, randomized study	21 female patients	Improvements in sleep quality, pain, fatigue, and depressive symptoms
Claustrat et al., 1997 [311]	Migraines	Open study	6 migraine patients and 9 healthy controls	Significant pain relief and absence of side effects

The MLT method of administration, phase of administration, and dosage are crucial factors that determine the efficacy of MLT. In most preclinical studies, the dosage of MLT used is many thousand-fold higher than the physiological endogenous MLT concentration, and it is probable that MLT may exert antinociception through different antinociceptive pathways depending on the dosage. In addition, the model adopted for studying neuropathic pain and evaluating central or peripheral neuropathic pain might result in different outcomes even if the same dosage is used [262].

Although the preclinical studies have shown MLT efficacy in different pain paradigms, several questions related to the mechanism of action remain unanswered. Despite the promising data obtained from studies conducted on animal models, there is a lack of clinical trials confirming the effectiveness of dietary MLT in alleviating or slowing down neuropathic pain. The inclusion of MLT-rich food in neuropathic patients’ daily diet may be a part of a multilevel nutritional intervention to prevent and better manage disability and quality of life. The MLT dietary source offers a lower-risk alternative to conventional treatments, which are often limited by side effects and suboptimal long-term efficacy. However, challenges remain in their routine clinical application, including ensuring consistent efficacy and developing standardized treatment protocols. Therefore, in the following paragraphs, we summarize the preclinical and clinical studies reported in the literature and discuss the possible mechanisms related to the pain relief of MLT dietary sources in different neuropathic states.

#### 4.1.2. Melatonin and Dietary Sources

Several studies have suggested that modifying dietary patterns by consuming certain nutrients can offer significant relief to people with neuropathic pain (Table 2). These interventions can improve neural functions with the help of drugs. We underline the fact that dietary and nutritional approaches could be important for the management of pain, together with pharmacological treatments [50,81,315,316].

Below, we summarize the dietary sources of MLT, considering its positive effects on inflammation, oxidative stress, and pain modulation [293].

##### Sources of Melatonin and Benefits of Consuming MLT-Containing Foods

Healthy eating is one of the most important pillars for health and quality of life, and several epidemiological and methodological studies have pointed to an association between healthy, balanced eating and improved health and quality of life.

The highest concentration of MLT can be found in fruit and vegetables [238]. Grapes, cherries, and strawberries are the most investigated fruits in terms of MLT content, even if they show differences depending on the harvest conditions and varieties [318]. A high content of MLT in vegetables has been found in tomatoes and peppers, while in potatoes, it is undetectable, and only a very low content can be found in beetroot [319,320].

MLT occurs in many legumes and seeds, such as white and black mustard seeds, and during germination, its content increases more than 11-fold [321]. Cereals such as corn and rice, nuts, and pistachios have been reported to have the highest MLT contents [322,323].

As MLT plays an important role in the physiological pathways, as described earlier, and its secretion decreases after childhood, raising dietary consumption is a good option. Several studies have shown that the consumption of foods rich in MLT increases its content in the blood, determining positive health impacts when the indolamine decreases [324,325]. Moreover, Sae-Teaw et al. (2013) found an increase in antioxidant capacities and MLT content in the serum of healthy male volunteers after the consumption of different fruits [325].

MLT is present in several types of food at different concentrations (ranging from picograms to milligrams/gram) [323,326]. It is important to underline that the MLT concentration in dietary sources may vary between tests, processing conditions, and the method of determination. It may also differ due to the dietary source being harvested from different trees or orchards, or in relation to the time of harvest (degree of ripeness) [247,327]. Most foods and drinks consumed by humans contain MLT, and their intake probably increases circulating MLT levels and the total antioxidant status of human serum [247,326]. In plants, MLT seems to function similarly to that in vertebrates: as a free radical scavenger and in photoperiodism [327,328]. Notably, in some plants, especially in flowers and seeds, MLT concentrations are several orders of magnitude higher than those normally measured in vertebrate tissues (except for the pineal gland) [328]. Hattori et al. (1995) investigated the oral MLT bioavailability from 24 edible plants and found that the administration of a diet consisting of plant products rich in MLT to 48 h fasted chicks significantly increased the level of circulating MLT (the daytime MLT levels were roughly doubled) [329]. The consumption of plant food, together with endogenous MLT synthesis, may modulate MLT blood levels; thus, dietary MLT may exert protective effects. Increasing circulating levels of MLT through dietary supplements thus intensifies its health benefits and generates no undesirable effects. Therefore, the health effects of diets rich in MLT-containing foods should not be underestimated.

Maldonado et al. (2022) showed that the MLT levels found in beer are low, in a range of pg/mL to ng/mL; however, the authors reported that MLT exerts its beneficial functions [247]. This is probably due to the fact that MLT can exert its functions directly by crossing the lipid cell bilayer and by saturating the MT1 and MT2 receptors. This means that low doses of MLT can be sufficient due to receptor affinities that are half-saturated in the physiological range of circulating MLT. Furthermore, it is possible to infer that an incremental rise in MLT blood levels may not be exclusively due to phytomelatonin intake from plants but may rather occur as a result of some plant constituents that trigger the release of endogenous MLT from the gut [247].

Aguilera et al. (2016), in an in vitro study, evaluated the effect of kidney bean sprout intake on the plasma levels of MLT and compared the plasma bioavailability derived from kidney bean sprouts versus synthetic MLT intake [330]. Notably, the authors reported slightly higher levels of plasmatic MLT (17%) in rats fed with the solution of synthetic MLT [330]. 

Burkhardt et al. (2001) pointed out that the consumption of cherries promotes anti-inflammatory and antioxidative effects in humans [327]. These effects may be related to the levels of MLT and/or other antioxidants contained in these fruits [327].

The implication of the previously reported data is that consuming food rich in MLT could be an important source of dietary MLT, inasmuch as MLT is readily absorbed when taken orally.

Therein, the use of dietary sources of MLT may be extremely beneficial in helping to maximize the health-promoting effects of medicinal plants and healthy foods in humans, possibly acting in synergy with other bioactive phytochemicals (i.e., polyphenols) that are ingested daily. However, the current lack of knowledge on the oral bioavailability of MLT in the human diet clearly indicates the need for more in-depth clinical trials. Future studies should consider the circadian and seasonal variations of endogenous MLT and estimate the amount of MLT ingested. In fact, as there is no difference between endogenously and exogenously acquired MLT, it is very difficult to assess the dietary contribution in humans [242].

The modern protein-abundant dietary pattern, rich in meat, poultry, fish, eggs, and milk, is associated with improved sleep quality. This is probably due to its high content of tryptophan and also of vitamin B12, which seems to aid in the regulation of sleep in addition to contributing to increased MLT synthesis and the number of its brain receptors [331].

MLT bioavailability presents an extensive variation, even when assessing its oral and/or intravenous administration. Furthermore, exogenous MLT pharmacokinetics, its half-life, and clearance are relatively uniform in laboratory animal models; however, in humans, a highly variable pharmacokinetic profile with low availability is reported. It easily crosses all morphophysiological barriers [242,326,332,333]. Furthermore, the intake of food sources of MLT significantly increases its circulating levels in humans [334]. Andersen et al. (2016) conducted a crossover cohort study to determine the pharmacokinetics of 10 mg of oral MLT or 10 mg of intravenous MLT in healthy male volunteers [335]. Orally administered MLT was rapidly absorbed, with the Tmax reaching 41 min. However, the Cmax and area under the curve (AUC) varied significantly among volunteers. Regarding oral and intravenous MLT elimination half-lives, they were 54 min and 39 min, respectively. Thus, oral MLT bioavailability was found to be only 3%, with considerable inter-volunteer variability [242,335]. These observations reflect the need for more in-depth studies on the pharmacokinetic properties of MLT and MLT dietary sources.

MLT, when consumed as a drinking fluid [336] or taken as a galenic tablet [337], is readily absorbed into the circulation. Thus, MLT from foods would also likely be absorbed. To date, MLT uptake from herbal remedies or products, and the oral bioavailability of phytomelatonin have not been completely explored. Yeleswaram et al.’s study, which assessed the oral bioavailability of synthetic MLT in rats, dogs, and monkeys, showed a dose-dependent availability that differed among the species examined [338].

MLT is metabolized mainly in the liver by hepatic cytochrome P450 (CYP) monooxygenases and conjugated to form 6-sulfatoxymelatonin, which is the main urinary metabolite of this indoleamine. In the brain, the primary metabolite is formed through oxidative cleavage, and the product is known as N1-acetyl-N2-formyl-5-methoxytryptamine (AFMK). This is demethylated either by arylamine formamidase or hemoperoxidases to form N1-acetyl-5-methoxykynuramine (AMK) [339]. The other metabolite of MLT is 3-hydroxymelatonin [340]. Interestingly, some MLT metabolites are even more potent than their precursor, such as 3-hydroxymelatonin (C3-OHM) or AMK [341,342].

As described earlier, MLT is a potent antioxidant molecule; however, it is important to highlight that, in the same fruits, there are other food components, such as polyphenols, that also have antioxidant properties [343,344]. Therefore, the beneficial effects of the consumption of food rich in MLT could be not only due to the MLT content but also the integration with other components that play a role in MLT metabolism, increasing its intake. The increase in MLT intake could contribute positively to sleep quality and antioxidant status [238]. This finding is important because it suggests the excellent role of MLT compared to other food components, stressing the beneficial functions of this indolamine.

### 4.2. Overview of Different Dietary Interventions Against Neuropathic Pain

Along with MLT, other nutrients may be beneficial in treating various painful conditions. Flavonoids, terpenoids, alkaloids, phenols, and carotenoids, as well as micronutrients such as vitamins and minerals, have been evaluated for their potential therapeutic effect in neuropathic pain models [49,345]. Several studies have focused on these nutrients because they all have anti-inflammatory properties. These studies have suggested that neuroinflammation plays a key role in chronic pain and that its regulation is a potential therapeutic approach [346,347].

In this paragraph, we summarize an overview of the studies published on different diet interventions for managing neuropathic pain. In detail, we focus our attention on the effects of the Mediterranean diet and nutraceutical diet interventions. This paragraph is strictly linked to the above-reported fundamental role of natural products (e.g., polyphenols, vitamins, and minerals) in treating neuropathic pain.

Dietary habits, influenced by lifestyle traditions, sociodemographic characteristics, and ethnicity, have a direct impact on people’s wellness and quality of life. However, there are still a few challenges to defining the health benefits of certain foods and diets, improving neuropathic pain, and reducing healthcare costs.

A meta-analysis showed that orally or topically administered polyphenol products could benefit patients with osteoarthritis by inhibiting the NLRP3 inflammasome and NF-kB pathways. In six randomized controlled trials, polyphenols reduced pain and improved functional outcomes compared to the placebo [348]. Valsamidou et al. (2021) reported that frequent consumption of food rich in polyphenols may reduce the prevalence of osteoarthritis [349]. In fact, quercetin, resveratrol, curcumin, and rosmarinic acid have been investigated for their antioxidant, anti-inflammatory, and chondroprotective effects. The proposed mechanism of action is twofold: the inhibition of apoptosis and the repair of articular cartilage damage [350].

Resveratrol is a natural polyphenol widely used for its cardioprotective and anti-aging properties. Recent studies have suggested that it could reduce neuropathic pain in an animal model in a time-dependent manner. Resveratrol relieves thermal hyperalgesia and allodynia more significantly when administered immediately after chronic constriction injury or at the peak of pain symptoms [351]. These effects may be attributed to the antioxidant and anti-inflammatory properties of polyphenols [352]. Tao et al. (2016) showed that resveratrol significantly reduced the mRNA expression of TNF-α, IL-1β, and IL-6 in the ipsilateral sciatic nerve of experimental mice compared to controls [351].

The Mediterranean diet has been recognized as a healthy dietary pattern in the Dietary Guidelines for Americans 2015–2020 from the US Department of Agriculture [353]. The Mediterranean diet consists of vegetables, fruits, cereals, and nuts, which provide key nutrients and fibers [354,355,356,357]. Olive oil, a monounsaturated fat, is the principal source of dietary lipids because of its high nutritional quality. Furthermore, eggs, legumes, white meat, and fish (seafood) are good sources of protein. Seed oil (sunflower, rapeseed, soya, or other seeds), a polyunsaturated fat, is used in many non-Mediterranean countries. However, these oils do not have the antioxidant capacity of olive oil, even though they are better than lard or butter [358,359].

In accordance with the strong positive correlation between the Mediterranean diet and general health, it is expected that a positive impact on neuropathic pain also exists. However, the Mediterranean diet is not the only “healthy diet”; other dietary patterns demonstrate a strong beneficial association with disease prevention. A Mediterranean-style diet with adequate micronutrients (omega-3, vitamin B12, and magnesium) coupled with a reduction in processed foodstuffs (meats and white flour products) is characterized by potential anti-inflammatory and antioxidant benefits for chronic pain patients, also reducing analgesic consumption [83,360]. Notably, it has been reported that neuropathic pain can be reduced by 40% with an anti-inflammatory diet [361]. The Mediterranean diet has been shown to be weakly associated with reductions in oxidative stress markers, as well as inflammation markers, while the vegan diet has shown a borderline reduction, suggesting lower systemic inflammation compared to omnivorous diets [82].

Minerals, particularly zinc and magnesium, are also used to treat painful conditions [49]. Several studies have reported that zinc depletion could be associated with increased prostaglandin levels and sensitization of nociceptive fibers; therefore, zinc could alleviate pain through its anti-inflammatory properties [362]. Zinc exerts its activity by suppressing substance P levels in FM patients [363] and through transient receptor potential cation channel subfamily V member 1 (TRPV1) inhibition in a paclitaxel-induced neuropathic model [364]. Magnesium, in contrast, has been suggested as a potential modulator of central sensitization and pain hypersensitivity [49]. Different studies have demonstrated that magnesium also reduces N-methyl-D-aspartate receptor (NMDA) sensitivity in rats [365,366]. This mechanism could be a promising therapeutic option for the management of painful conditions [137].

Klowak et al. (2024), in a recent systematic review series, reported the role(s) of lifestyle interventions (such as diet, physical activity, health counseling, and mindfulness-based stress reduction) in the management of neuropathic pain [71]. Recent randomized controlled trials have indicated that dietary lifestyle interventions have the potential to reduce the subjective and objective burden of neuropathic pain in a variety of affected patient populations [71]. However, there is a significant lack of large and comprehensive trials assessing dietary lifestyle interventions within specific populations reporting the same outcomes. Torlak et al. (2022) reported that a plant-based fasting-mimicking diet with a Mediterranean diet infrequently improves neuropathic outcomes [367]. However, a low-fat, plant-based diet, a low-calorie diet, and a potassium-reduced diet may confer positive and beneficial neuroprotective and anti-inflammatory effects that reduce neuropathic severity overall [368,369,370]. In detail, a potassium-reduced diet may be appropriate for a patient population affected by chronic kidney disease; however, in subjects with normal renal function, it may be deleterious. As such, the findings of that particular trial are unlikely to generalize as broadly as those of the assessed interventions that would be safe across large populations of patients with neuropathic pain, regardless of etiology (e.g., a whole-food, plant-based diet) [124]. Additionally, given the plentiful and complex pathways that diet and dietary constituents can influence, it is difficult in such trials to disentangle the causal relationships hypothesized in the available literature [71].

Bunner et al. (2015) conducted a randomized controlled pilot study on 35 patients affected by type 2 diabetes and with a diagnosis or symptoms of painful diabetic neuropathy for at least 6 months [370]. These patients were submitted to diet interventions, omitted animal products, limited fat intake to 20–30 g per day, and favored low-glycemic-index foods. The diet focused on vegetables, fruits, grains, and legumes. After 20 weeks of the diet, the patients reported relief of pain [370]. The mechanism(s) by which the low-fat plant-based diet improves neuropathy pain may involve improved insulin sensitivity, leading to better glucose control [371]. In addition, diabetic neuropathy is associated with hypertension, dyslipidemia, and obesity, all of which have been shown to be ameliorated with a plant-based diet [372,373,374].

Castañeda-Corral et al. (2021) showed that the cafeteria diet, along with low doses of streptozotocin, in mice can be effectively used to generate an easily accessible, fairly economical, and reliable model of peripheral neuropathy associated with type 2 diabetes mellitus after the inducement of obesity and β cell impairment in female C57BL/6J mice [375]. The cafeteria diet consists of highly palatable and energy-dense foods, which are very common in Western society. This model mimics some of the metabolic features, mechanical allodynia, and a reduction in cutaneous innervation reported in humans affected by type 2 diabetes mellitus. Thus, this preclinical mouse model could be a useful and translational tool in evaluating the efficacy of new drugs to treat painful diabetic neuropathy and improve translation [84].

Borgonetti and Galeotti (2022) investigated the in vitro and in vivo senolytic activity of rosmarinic acid and its potential benefit in managing peripheral neuropathic pain symptoms in a spared nerve injury model [376]. Rosmarinic acid is known for its antioxidant, antibacterial, antiviral, anti-inflammatory, analgesic, neuroprotective, cardioprotective, and many other activities [377]. Notably, the authors reported that rosmarinic acid could be quickly absorbed into the blood and eliminated slowly [378], and it is able to control both hyperalgesia and behavioral disturbances. The cellular aging process develops in affected tissues during neuropathy, and rosmarinic acid could reduce inflammation and microglial senescence in vitro. All of the above-reported rosmarinic acid effects present important pharmacological advantages in managing peripheral neuropathy.

In recent years, the great availability of nutraceuticals has drawn considerable attention from patients seeking relief from chronic pain conditions. Nutraceuticals, intended as dietary supplements and herbal/natural products, seem to have beneficial effects in the treatment of neuropathic pain due to their ability to bind different molecular targets [49]. For example, flavonoids exhibit important antioxidant activity and the capacity to modulate protein kinase C [345].

**Figure 12:**
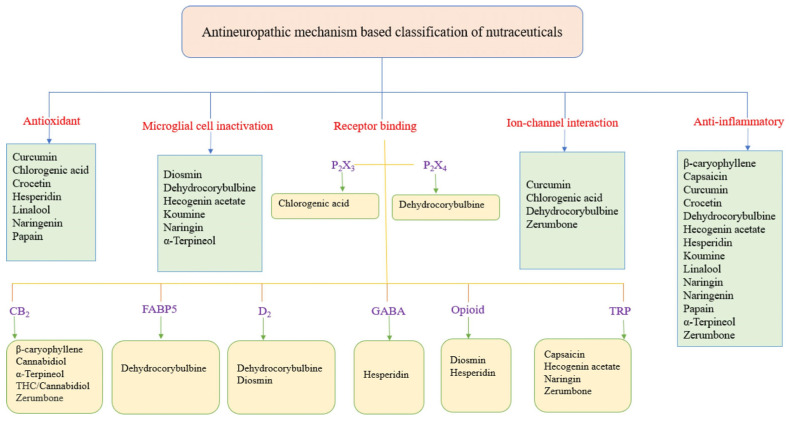
Antineuropathic mechanisms of action of nutraceuticals. Illustration from Mishra et al. [49] (license number 5997650825107).

Mishra et al. (2022) identified oxidative stress, inflammation, ion channels, and microglia as primary targets for neuropathic pain treatment [49]. Figure 12 summarizes the main mechanism of action of nutraceuticals.

Select novel drugs for FM, such as palmitoylethanolamide, an endogenous endocannabinoid mediator commercialized as a dietary food for special medical purposes, downmodulate the activation of mast cells and microglia and have anti-inflammatory and antihyperalgesic properties [379]. A recent randomized placebo-controlled study on healthy volunteers highlighted that palmitoylethanolamide reduces peripheral and central sensitization while also increasing pain modulation [380]. Furthermore, a new generation of nutraceuticals has been formulated, with synergistic action, as a useful adjuvant in managing FM symptoms. This innovative formula, rich in palmitoylethanolamide, alpha-lipoic acid, and Gynostemma pentaphyllum, helps neural and synaptic trophism, reduces oxidative stress, and combats the symptoms of asthenia [379].

Piperine, a component of black pepper extract, also shows significant analgesic and neuroprotective efficacy with potential additive efficacy in managing FM [381,382].

Additionally, curcumin has emerged as a promising candidate for the treatment of neuropathic pain in recent years [49,383]. Additionally, accumulating evidence shows that chlorogenic acid exhibits antinociceptive action in several neuropathic models. In a chronic constriction injury-induced neuropathic pain model, chlorogenic acid significantly attenuated the development of mechanical hyperalgesia [384]. Furthermore, this phenolic compound has a strong antinociceptive effect in diabetic neuropathy animals [385].

Vitamins and minerals are essential micronutrients for the healthy development of the body. Several studies have shown that vitamins B and D could alleviate neuropathic pain, particularly diabetic neuropathy [386,387]. The vitamin B complex is known to have neuroprotective activity through the activation of astrocytes and microglial cells and increased GABA synthesis or modulation of TRPV1 [137]. The analgesic effect of vitamin B itself is not strong; thus, a synergistic mechanism with anti-inflammatory agents is required [137]. Recent studies have demonstrated that vitamin D may reduce inflammation and the risk of several chronic diseases, such as cardiovascular diseases, autoimmune diseases, diabetes, and multiple sclerosis [49]. Therefore, vitamin D supplementation in patients with a deficiency might be associated with pain relief [49]. Huang et al. (2013) found that standardized vitamin D supplementation in veterans with multiple areas of chronic pain may effectively improve pain levels, sleep, and various aspects of quality of life [388].

Jolivalt et al. (2009) showed that repeated application with a cocktail of B vitamins improved tactile allodynia and formalin-induced hyperalgesia, along with sensory nerve conduction velocity in diabetic rats [386]. In another study, B1, B6, and B12 vitamins, alone or in combination with carbamazepine, ameliorated nociceptive pain behaviors in a trigeminal neuropathic pain model [389]. Furthermore, a clinical study demonstrated that an orally administered fixed dose combination of methylcobalamin, α-lipoic acid, folic acid, biotin, benfotiamine, and vitamin B6 capsule resulted in a significant reduction in peripheral neuropathic symptoms with no evidence of adverse reactions [390].

Nutrients such as magnesium, vitamin B12, and zinc have been shown to reduce hyperalgesia and allodynia in animal models of neuropathic pain induced by nerve injury through actions on N-methyl-D-aspartate receptors [391,392,393]. Magnesium produces antinociceptive effects in animal models of neuropathic and inflammatory pain [394], and magnesium supplementation has been shown to alleviate acute postoperative and chronic neuropathic pain and enhance the effect of opioids without a concurrent increase in side effects [392,395].

The administration of gallic acid is also able to decrease cold and mechanical allodynia in the neuropathic pain model [396].

As more consumers use nutraceuticals for disease prevention [397], their efficacy as therapeutic agents is determined by different pathways.

Ilari et al. (2022) reported a systematic review and meta-analysis of the existing literature on animal studies, in which they examined the mechanisms of action of different nutraceuticals in treating neuropathic pain and assessed the influence of confounding factors [398]. The authors reported that the administration of natural drugs improved thermal hyperalgesia regardless of the type of animal, the behavioral test, or the route of administration. Furthermore, the analysis showed that natural drug administration improved mechanical allodynia/hyperalgesia symptoms primarily in chemotherapy or diabetes-induced neuropathic pain (although only four studies used these mechanisms) compared to mechanically induced neuropathic pain (15 studies) [398].

Further studies are needed; however, improving dietary habits could play a crucial role in optimizing neuropathic pain relief and related outcomes.

## 5. Limitations

Although the potential therapeutic effects of MLT have been well demonstrated in animal models, published clinical data remain limited, partly due to the relatively early stage of research into its novel therapeutic applications. Future studies involving human participants are essential. Furthermore, concerns have recently been raised regarding the administration of MLT at higher doses to ensure its efficacy [399]. It should be noted that its inclusion in the Mediterranean diet, as discussed in the previous paragraphs of this review, could help to reinforce these concepts and advance this perspective.

## 6. Conclusions

In conclusion, the management of neuropathic pain and related disorders requires multimodal and multidisciplinary approaches, as reported by Badaeva et al. (2023) [235]. A synergistic effect is possible through collaboration among clinical specialists, nutritionists, and other professionals, considering the patients’ comorbidities. Nutritional neuroscience suggests that a personalized approach combining nutrition, diet, exercise, and other therapies could complement clinical management.

Given the significant roles of MLT in metabolic pathways, we emphasize the potential benefit of increasing the dietary intake of this indoleamine, along with other antioxidant and anti-inflammatory substances, as a complementary approach to pharmacological therapies in modulating or reducing neuropathic pain.

## Figures and Tables

**Table 2:** Nutritional diet and neuropathic pain. Table from Cuomo and Parascandolo [50].

Intervention	Outcome	Study
Nutritional changes (dietary patterns, nutrients, meal frequency)	Relief comparable to antidepressants, improvement in musculoskeletal conditions	[81,315,316]
Mediterranean diet	Alleviates pain, reduces stiffness, and addresses biomarkers in osteoarthritis	[317]
